# SPICE Model for SiC Bipolar Transistor and TTL Inverter Degradation Due to Gamma Radiation

**DOI:** 10.3390/mi16111246

**Published:** 2025-10-31

**Authors:** Alex Metreveli, Anders Hallén, Carl-Mikael Zetterling

**Affiliations:** Division of Electronics and Embedded Systems, School of Electrical Engineering and Computer Science (EECS), KTH Royal Institute of Technology, Teknikringen 31, 100 44 Stockholm, Swedenbellman@kth.se (C.-M.Z.)

**Keywords:** SiC, SPICE, BJT, transistor, radiation, gamma radiation, IC, inverter

## Abstract

Silicon carbide (SiC) is a key material for electronics operating in harsh environments due to its wide bandgap, high thermal conductivity, and radiation hardness. In this work, we present a SPICE model for a 4H-SiC BJT and TTL inverter exposed to gamma radiation. The devices were fabricated using a dedicated SiC bipolar process at KTH (Sweden) and tested at the ^60^Co Calliope (Italy) facility up to 800 krad _(Si)_. Experimental data, including Gummel plots and inverter transfer characteristics, were used to calibrate and refine a VBIC-based SPICE model. The adjusted model accounts for both bulk and surface degradation mechanisms by extracting parameters of forward current gain (βF), saturation current (IS), base resistance (RB), and forward transit time (TF). Results show a uniform degradation of BJTs, primarily manifested as reduced current gain and increased base resistance, while the inverter maintained functional operation up to 600 krad_(Si)_. Extrapolation of the SPICE model predicts a failure threshold near 16 Mrad_(Si)_, far exceeding the tolerance of conventional silicon circuits. By linking radiation-induced defects at the material and interface levels to circuit-level behavior, the proposed model enables realistic design and lifetime prediction of SiC integrated circuits for satellites, planetary missions, and other radiation-intensive applications.

## 1. Introduction

In this study, we develop a SPICE model for 4H-SiC-based transistor–transistor logic (TTL) bipolar junction transistor (BJT) inverters that are built to handle high gamma radiation levels. Silicon carbide (SiC) is an attractive material for these applications because of its wide bandgap, excellent thermal conductivity, and high electrical field tolerance—features that make it a great candidate for use in aerospace and high-temperature electronics. For a broader review of SiC device applications under harsh environmental conditions, see, for example, [1,2].

An inverter carries out a basic, but crucial, logic operation called a “complementary”, or “NOT” operation, which flips an input signal from 0 to 1 or vice versa. Such simple action is key to constructing more complex logic functions. However, logic and integrated circuits (ICs) that are used in extreme environments like outer space, where radiation levels are high, might act very differently compared to their behavior under normal operating conditions. This distinction between device- and circuit-level responses under total ionizing doses has been discussed in depth in [3,4]. Specifically, the way an inverter reacts to radiation can be quite different from how a single transistor responds [5], highlighting the need for deeper investigation into these differences. Typically, a large number of expensive and time-consuming experiments are needed to assess the impact of different radiation levels on the SiC bipolar ICs, and therefore this research area is not very well-studied [6,7]. In the present work, we propose a way forward to study the impact of gamma irradiation on any SiC-based bipolar IC, without the need for additional experiments, through presenting a SPICE model of SiC bipolar transistors under a wide range of radiation conditions.

The core of this research is about fine-tuning a SPICE (Simulation Program with Integrated Circuit Emphasis) model for BJT and TTL inverters. The findings are not just useful for space tech, but also have applications in nuclear facilities and medical equipment, where electronic components must operate reliably under radiation exposure. The SPICE model helps us to better understand and enhance the durability and performance of SiC NPN inverters, showing that they can be a more reliable choice compared to traditional silicon-based options, and explain their behavior. This study takes a practical step towards improving electronics that can endure some of the toughest environments, both on and beyond Earth. Building on prior work in this domain [5,8,9], our study significantly extends understanding of SiC bipolar circuit low-power ICs. Although silicon carbide (SiC) is known for its advantages in high-temperature and radiation-rich environments [9,10,11,12], the literature on modeling SiC TTL inverters for such extreme conditions remains fragmented. Introducing SPICE models for SiC transistors under gamma radiation exposure addresses existing research gaps, offering a new perspective on designing radiation-hardened integrated circuits.

It is demonstrated that the effect of gamma rays is much lower for these chips than for corresponding Si circuits. Using our verified SPICE model shows that the inverter should be in operation even for doses far exceeding 16 MRad_(Si)_.

The choice of the BJT structure over the more common Metal-Oxide-Semiconductor Field-Effect Transistor (MOSFET) is motivated by two key factors. First, SiC BJTs are inherently more resistant to radiation than their MOSFET counterparts [13,14]. Their bipolar structure relies on minority carrier injection (diffusion current) and does not depend on an inversion channel at the SiC/SiO_2_ interface, which is a critical point of failure in MOSFETs due to radiation-induced charge trapping [3,13,14]. Second, this specific research builds upon a well-established and mature SiC bipolar process technology available at KTH [5,6,10], allowing for robust device fabrication and comparative analysis with existing data. Therefore, the BJT platform provides a superior foundation for developing radiation-hardened integrated circuits.

## 2. Experimental Section

### 2.1. Device Processing and Design

#### 2.1.1. Discrete 3-Pad Devices

The SiC bipolar devices and integrated circuits were fabricated at the MyFab Electrum Laboratory, located at the KTH Royal Institute of Technology in Kista, Sweden. The manufacturing process adhered to a procedure that replicates the steps outlined in the referenced process descriptions [8,9,15,16].

**Figure 1:**
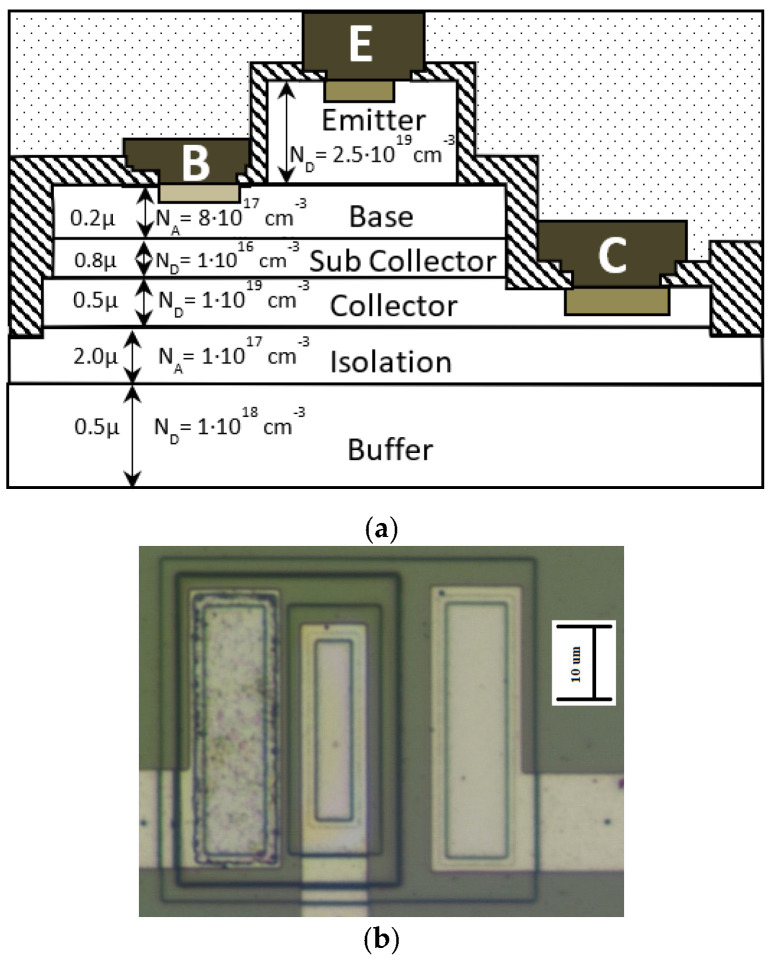
(**a**) The cross-section of the device (not scale), with the substrate and epitaxial layers’ cross-sections; (**b**) a top-view of the transistor contact layout.

The initial material used for processing was 100 mm, 4° off-axis, 4H-SiC n-type conducting wafers from Cree^TM^. These wafers feature multiple epitaxial layers that were grown in a single sequence to reduce interface states between the layers. The cross-sectional structure of a single transistor is depicted in Figure 1.

The first epitaxial layer (bottom), known as the buffer layer (0.5 µm, N_D_ = 1∙10^18^ cm^−3^), is utilized to minimize defect formation during epitaxial growth. Subsequently, the p-type isolation layer (2 µm, N_A_ = 1∙10^17^ cm^−3^) serves to effectively isolate individual devices, owing to its substantial p-type doping concentration. The subsequent layers adhere to a standard design for low-voltage bipolar transistors: an n+ collector for contacts (0.5 µm, N_D_ = 1∙10^19^ cm^−3^), a lower-doped sub-collector that blocks voltage (0.8 µm, N_D_ = 1∙10^19^ cm^−3^), a medium-doped p-base (0.2 µm, N_A_ = 1∙10^17^ cm^−3^), and a highly doped n+ emitter (2 µm, N_D_ = 2.5∙10^19^ cm^−3^) to complete the structure. The buried collector layers are positioned to establish low-resistance ohmic contacts with the lightly doped collector layers situated above them. The doping level and thickness of the base layer plays critical role in determining the device’s gain, while the lightly doped collector layers are influencing the device’s breakdown voltage. The top layer, the highly doped emitter layer, significantly enhances emitter injection efficiency and simultaneously reduces contact resistance, contributing to the overall performance of the device. The NPN transistors undergo a fabrication process involving three dry-etching steps, each designed to isolate the emitter, base, and collector regions, respectively. In the context of this research, it is important to clarify that a sacrificial thermal oxidation step is undertaken to remove approximately 10 nm of damaged SiC resulting from the dry-etching process. The entire surface is then passivated by a PECVD oxide layer, which is subsequently annealed in a N_2_O environment (1100 °C, 5 h). The interconnections are made from TiW to increase the temperature tolerance of the devices [5] and investigate the radiation response of the interconnected materials. The thickness of the metal is 2 µm, processed in 2 steps.

#### 2.1.2. Inverter Design

**Figure 2:**
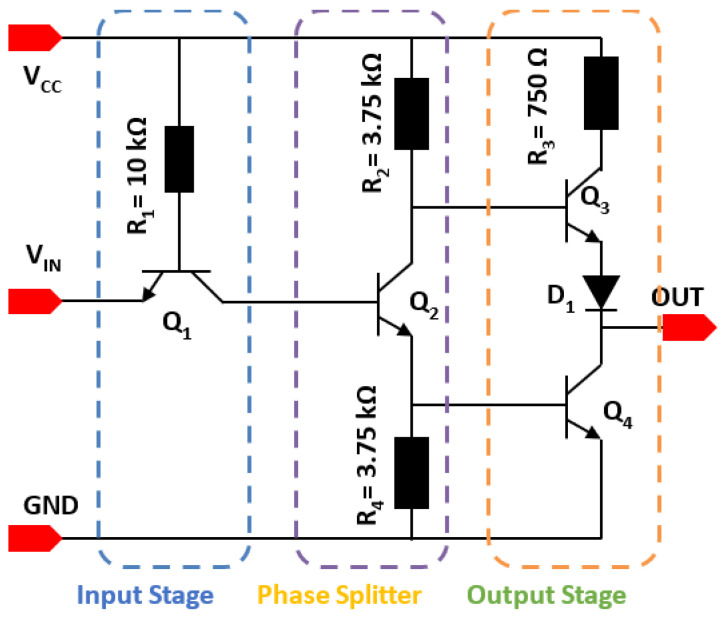
Schematic of the SiC TTL inverter.

**Figure 3:**
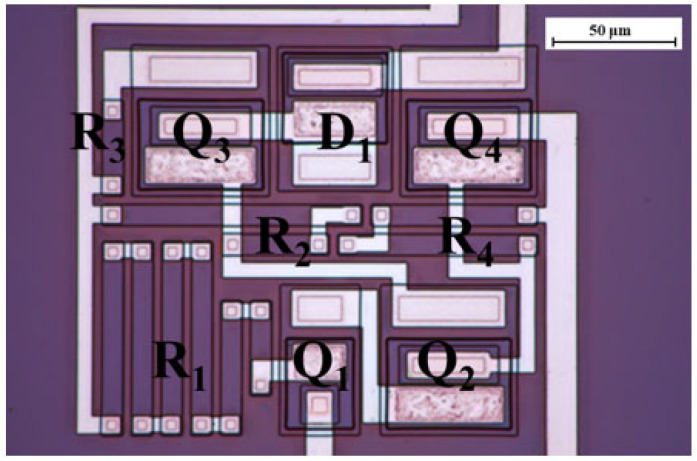
Corresponding layout of SiC inverter.

The transistor–transistor logic (TTL) inverter is a foundational component in digital integrated circuits (ICs), essential for manipulating logical states. Due to the inherent TTL architecture, only NPN transistors are required for its implementation. The schematic of the designed TTL inverter, implemented with 4H-SiC BJTs, is depicted in Figure 2 and Figure 3 [16]. This circuit comprises three primary stages characteristic of standard TTL configurations:

1. First Stage (Input Gate—Transistor Q_1_): This stage functions as the input gate. In this design, an emitter transistor Q_1_ is utilized to efficiently manage incoming signals.
2. Second Stage (Phase Splitter—Transistor Q_2_): This stage’s primary role is to invert and split the phase of the logic signal received from the first stage, preparing it for the final output stage.
3. Third Stage (“Totem-Pole” Output Configuration—Transistors Q_3_ and Q_4_): This crucial stage is implemented as a “totem-pole” output configuration, comprising two transistors (Q_3_ and Q_4_) that operate in a complementary fashion. This design ensures low output impedance in both high- and low-logic states. For instance, in the logic-high output state, transistor Q_3_ is “ON”, while Q_4_ is “OFF”, thereby driving the output to the desired logic level. Conversely, for a logic-low output, Q_3_ is OFF and Q_4_ is ON.
4. Diode Representation: In the schematic, the diode, typically present in TTL for level shifting, is replaced by an NPN transistor with its collector shorted to the base. This configuration ensures that the diode voltage drop precisely matches the base–emitter voltage drop of the subsequent transistor, optimizing signal levels.

Figure 2 also presents the corresponding layout (top-view) of the SiC TTL inverter, illustrating the physical arrangement of the components on the chip.

### 2.2. Radiation Testing Facility and Conditions

Gamma radiation experiments were conducted at the ^60^Co Calliope facility [17] with a photon mean energy of 1.25 MeV. A key advantage of this facility is that materials tested here do not become radioactive, allowing for immediate handling after irradiation. To ensure precise radiation dosing, we used the ESR–alanine dosimetry method. To combat issues like Compton backscattering from the lower-energy photons in the ^60^Co spectrum, we added extra shielding, which included a 2 mm thick aluminum box topped with a 2 mm aluminum/lead lid to protect the devices under test (DUTs).

The devices were tested across a range of gamma ray doses and dose rates, employing an aluminum/lead (Al/Pb) filter to adjust the doses to the expected radiation conditions in space. The radiation dose rate was maintained at 9.975 rad_(Si)_/s throughout the experiments at the DUTs.

Due to high energy of the photons and an activity of approximately 10^13^ Bq in the facility, we had to use long (18 m) lines in a 4-wire scheme to save the supply and measurement equipment. The experiment was performed under normal environment conditions. All measurements were conducted at a temperature of approximately 21 °C.

Since the project was carried out as part of space research, the following test doses presented in Table 1 are considered reasonable.

Thus, a dose of 800 krad_(Si)_ sufficiently covers (with an operational time and safety margin) the majority of missions that are not aimed at studying the sun and near-solar space.

### 2.3. Measurement Procedure

The testing of the devices was in adherence to semiconductor industry standards (MIL-STD-883L and MIL-STD-750 (Method 1019.9)) [18,19]. We assembled the setup such that these tests were performed in real-time with a remote setup that enabled both operation and immediate in situ characterization post-exposure. This method provides a clearer picture of gamma radiation’s impact compared to previous approaches [5,6].

We conducted electrical measurements using National Instruments^TM^ equipment, taking special care to shield against radiation-induced noise and maintain calibration accuracy. The system comprised an NI PXIe-1078 chassis, a PXIe-4081 digital multimeter (DMM), two PXIe-4136 source-measure units (SMUs), a PXI-4110 programmable DC power supply, and an NI PXIe-2532 switching matrix. Due to the presence of a radiation field in proximity to the device under test (DUT), measurements were conducted remotely. We utilized 18.7 m shielded cables to minimize noise interference. Our protocol ensured that exposure times were always ten times longer than measurement durations to improve the reliability of our results. We documented the direct current (DC) characteristics of the SiC BJTs, keeping a steady V_CC_ of 15 V and varying V_IN_ from 0 to 15 V. We also implemented a trimmed mean method to cut down on noise, which was particularly effective given the long cable lengths in our setup. To ensure measurement accuracy, each voltage and current value was sampled seven times. Subsequently, the highest and lowest 15% of the samples were discarded, and the mean of the remaining values was calculated. This averaging technique facilitated the measurement of low currents despite the use of extended cable lengths. Due to the remote measurement configuration and the implementation of quality control procedures, the duration of each test iteration, following radiation exposure, was 2 min. The radiation exposure time was set to be at least ten times greater than the measurement time, maintaining a critical 10:1 ratio between exposure and measurement periods.

The system was programmed to perform standard DC characterization tests, including Gummel plots and measuring I_C_-V_CE_ output characteristics. For the Gummel plot measurements, the collector–emitter voltage (V_CE_) was held constant at 15 V, while the base–emitter voltage (V_BE_) was swept from 0 V to 15.6 V. Due to constraints imposed by the radiation environment and the use of extended cabling, a relatively large V_BE_ step size of 0.2 V was employed. A current compliance limit was enforced at 1 A for each terminal, with automatic adjustment capabilities to ensure adequate current resolution.

## 3. Results

### 3.1. Experimental Results from SiC BJT

Bipolar transistors, by their design properties, show higher radiation hardness compared to MOSFET devices [20,21]. This distinction arises from the specific mechanisms of current flow and voltage application in the crystal structure. While the basic mechanisms are presented in [21,22,23,24], this work focuses on the practical aspects of transistor behavior. In operational scenarios, transistors are predominantly used in the amplification mode (common-emitter configuration), or as logic switches. More detailed discussions are presented in [8,9]. The inverter behavior and experimental results are discussed in [25].

**Figure 4:**
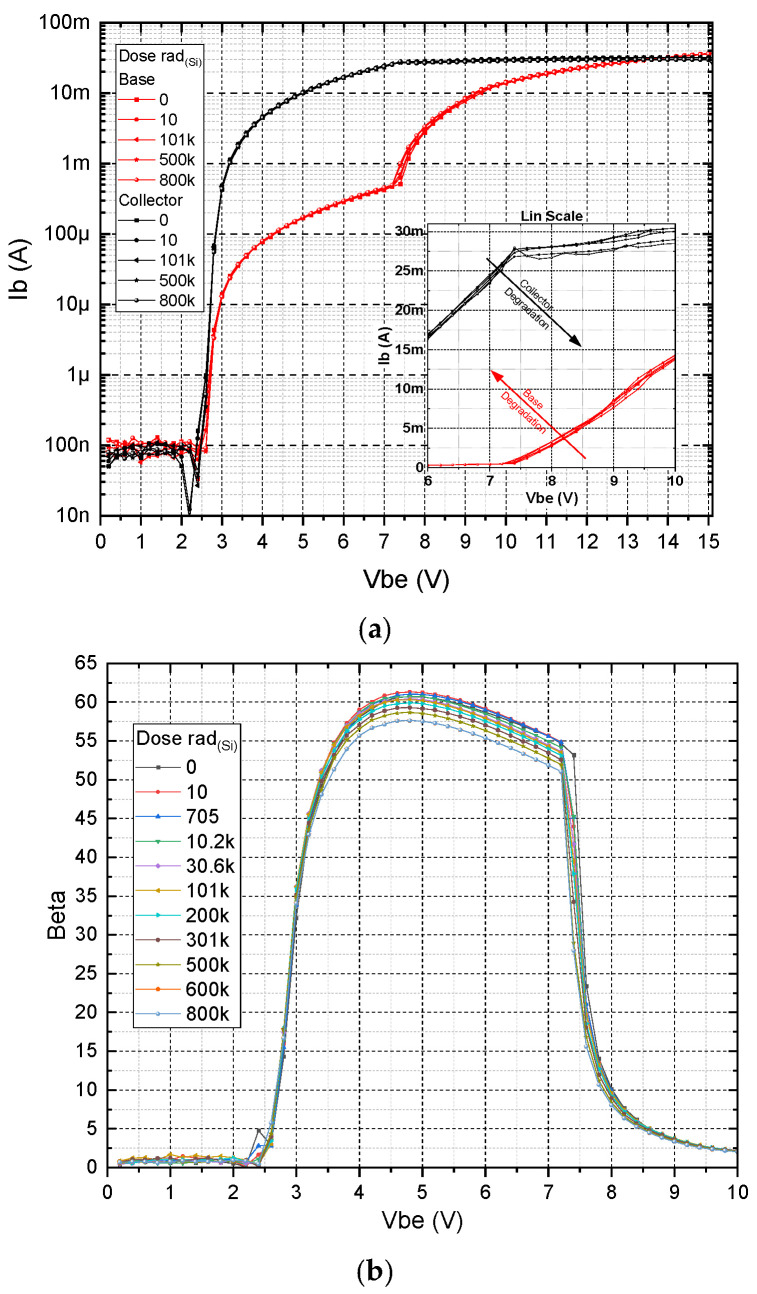
(**a**) Typical experimental Gummel plots for an integrated SiC BJT under various gamma doses. (**b**) Measured amplification factor β as a function of base–emitter voltage for increasing gamma ray exposure time.

In Figure 4 the measured Gummel plot and amplification β of a typical transistor in our inverter as a function of increasing gamma exposure up to 800 kRad_Si_ are shown. The dose rate for this measurement was kept at 9.975 rad_(Si)_/s. As can be seen, the effect of the radiation can barely be seen on the logarithmic Gummel plot (Figure 4a), but the change in the amplification, although small, is clearly distinguished on a linear scale (Figure 4b).

The general characteristics of the devices align, to a large extent, with those of silicon-based transistors. However, for our SiC-based devices, due to the higher bandgap energy E_g_, the TURN-ON processes start at higher voltages and there is a higher impact of base current observed in the high base–emitter voltage range. This phenomenon can be attributed to the geometric ratio between the emitter and base regions [26]. However, the observed characteristics enable the attainment of optimal base-to-collector current ratios, serving as a critical metric of transistor amplification efficiency. Since it is difficult to determine and analyze the data, and also because the main parameter/criterion is the current amplification β, we will look at the β characteristics in a separate figure.

The onset of base current dominance, where the collector current approaches saturation, is identified as an inherently unstable operating region. This variability is strongly influenced by deviations in the geometric accuracy of the transistor structure, which is determined by the quality of fabrication processes. Particular attention should be given to the slope of the collector top and the wall profiles of the transistor regions, as these parameters critically affect base performance.

### 3.2. SPICE-Simulated SiC BJT Characteristics

For the simulation of device characteristics, the NI Multisim v. 12 was used, which is built upon an advanced SPICE simulation engine. The VBIC (Vertical Bipolar Inter-Company) BJT [27,28] model was selected as it provides a comprehensive framework that includes parameters to account for non-ideal effects such as the Kirk effect at high injection levels and recombination phenomena at the emitter and collector junctions. These factors are essential for the modeling of SiC devices.

To ensure the accuracy of our simulations, the VBIC model’s parameters were adjusted to align with the initial experimental data from our non-irradiated SiC BJTs. This initial calibration allowed us to establish a baseline model that accurately represents the experimental characteristics of the real devices. The extracted parameters from this process are provided in Appendix A. This calibrated model then served as a foundation for predicting the effects of very high gamma ray irradiation.

#### 3.2.1. Initial SPICE Model Calibration

**Figure 5:**
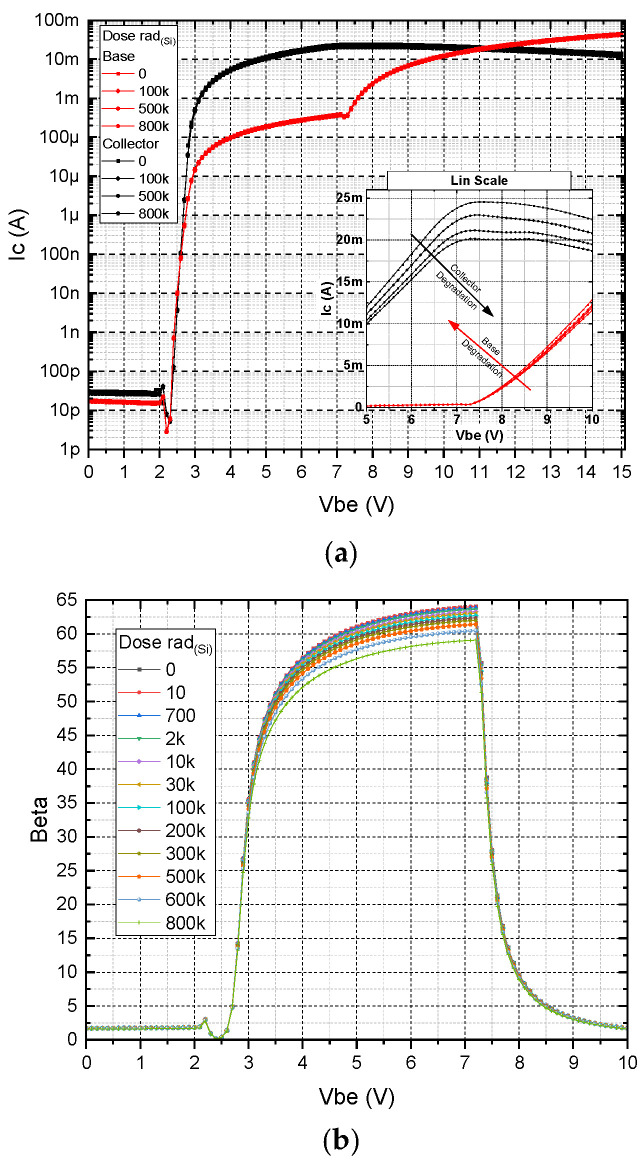
(**a**) Simulated ideal Gummel plot for SiC BJT under various gamma doses. (**b**) Simulated amplification factor β as a function of base–emitter voltage for increasing gamma ray exposure time.

Figure 5 presents the characteristic behavior of the base and collector currents for the ideal SPICE-simulated SiC transistor for gamma doses up to 800 kRad_Si_.

Under ideal conditions, the dynamic behavior of the base and collector currents aligns closely with simulated results, demonstrating agreement in both the linear and saturation regions of operation. But with an extra part in the higher voltages 5.0–7.1 V it still increases, with a fast ramp-down after 7.1 V due to the boost of *I_b_*, causing a rapid drop in β at voltages higher than 7.1 V.

When comparing the β behavior for experimental and simulated SiC BJTs, shown in Figure 4b and Figure 5b, it is also evident that the there is a clear deviation at a *V_be_* of about 5 V, where the measured values begin to drop while the simulations show an increased β. Ideally, beta should grow until the base current sharply overlaps the collector current.

The observed divergence in beta profiles at peak values is primarily attributable to common model imperfections, particularly the omission of coefficients accounting for interface irregularities and recombination losses at junction boundaries.

#### 3.2.2. SPICE Adjustment

We focused on extracting both bulk- and surface-related [28,29,30] SPICE parameters: the saturation current (*IS*), the emission coefficient (*NF*), the surface generation current (*ISE*), and its corresponding ideality factor (*NE*). Additionally, the static current gain β was calculated for each dataset. The total collector current was modeled using a nonlinear fit of the following expression:

$$Ic\;=\;IS\;\times \exp\left(\frac{Vbe}{NF\;\times \;VT}\right)+\;ISE\;\times \exp\left(\frac{Vbe}{NE\;\times \;VT}\right)$$

where *VT* is the thermal voltage at 21 °C.

This equation is based on a modified version of the Gummel–Poon model [27] and its modification [28] and adjusted for two primary contributions to the total collector current, each corresponding to a distinct physical phenomenon:

*Bulk Component:* The first term, arising from Compton scattering, describes the ideal diffusion current through the bulk semiconductor region. Here, *IS* is the ideal saturation current and *NF* is the emission coefficient, which deviates from unity to account for non-ideal recombination effects within the bulk.

*Surface Component:* The second term is included to model non-ideal recombination currents at the device’s surfaces and interfaces, particularly at the emitter–base junction’s sidewalls. *ISE* represents the saturation current associated with these surface effects, while *N_E_* is the corresponding ideality factor. This component becomes especially significant under conditions where surface degradation and trap generation are prominent, such as after exposure to ionizing radiation.

The use of this dual-exponential model is crucial for our study as it provides a framework for disentangling the effects of bulk and surface degradation. By fitting our experimental data to this model, we can individually track the changes in parameters like *IS*, *NF*, *ISE*, and *NE*, thereby gaining deeper insight into the physical mechanisms of radiation-induced degradation in the SiC BJTs. This approach allows for a precise analysis of how radiation-induced defects in both the semiconductor bulk and at the SiC/SiO_2_ interface affect device performance.

**Figure 6:**
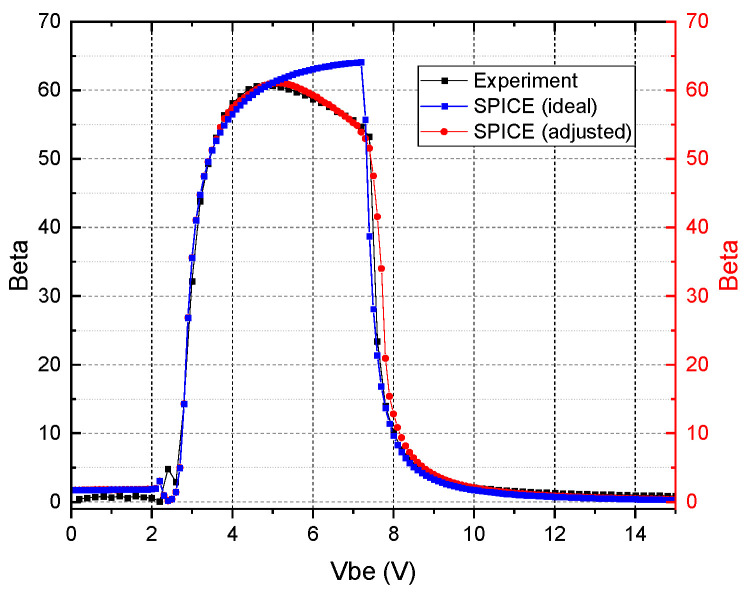
Comparison of ideal vs. experimental beta curves before radiation exposure.

In Figure 6, we compare the β values of the SiC BJT before exposure to the “standard”, as well as the adjusted SPICE models.

#### 3.2.3. Comparing SiC BJT Measurements and Initial and Adjusted SPICE Models

**Figure 7:**
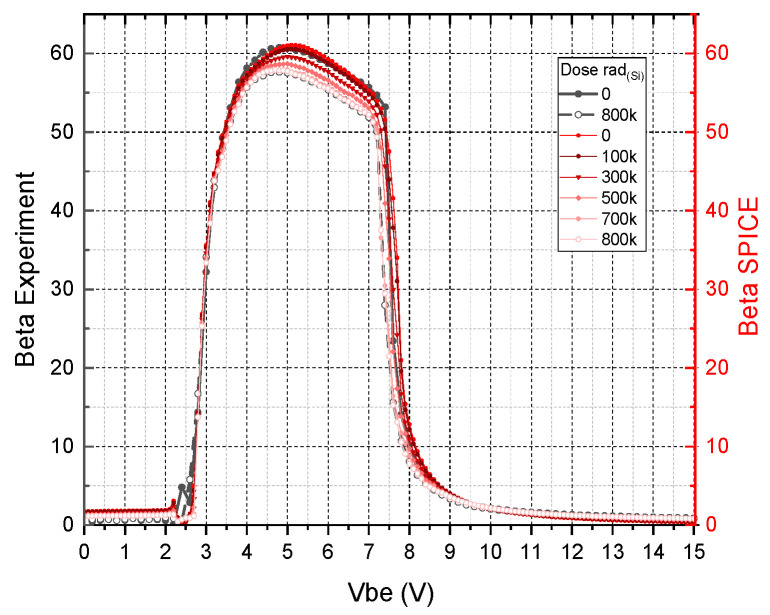
Degradation of beta during radiation exposure: experimental vs. (adjusted) SPICE model.

To understand the specifics of the degradation itself, its mechanisms and modes, we will refer to the work in [8]. It should be noted that the degradation is uniform. This paper presents the beta characteristics for the initial point (before irradiation) and the final irradiation point (800 krad_(Si)_). The beta degradation plots are presented in Figure 7, where experimental curves are shown in gray, while SPICE-modeled beta characteristic curves for different doses are shown in various shades of red.

The comparison between experimental and SPICE model results underscores the challenges associated with developing accurate circuit-level models. The divergence in beta characteristics reflects the natural variability inherent in experimental conditions. Specifically, the initial beta increase (observed in the first measurement points) can be attributed to induced noise within the high-radiation environment, which affects both the DUT and associated transmission lines.

Despite these challenges, the results validate the robustness of the proposed model for engineering evaluations. The outcomes demonstrate the suitability of the approach for predicting transistor behavior under radiation exposure, offering valuable insights for the design of radiation-hardened integrated circuits.

To effectively design integrated circuits, accurate circuit-level models are essential for the included components. Given that the current gains and collector resistances of SiC BJTs can vary widely based on their design and manufacturing processes, it is necessary to develop a custom model or a series of models tailored to the observed electrical behaviors of the BJTs in question. When incorporating a newly designed BJT, the methodology depends on calibrated physical device simulations that forecast the electrical characteristics of the BJT.

The accuracy of the models is important for the design of analog and digital circuits, especially when specific criteria for power consumption are to be met; thus, selecting appropriate reference devices and accurately calibrating the simulation models are vital steps.

**Figure 8:**
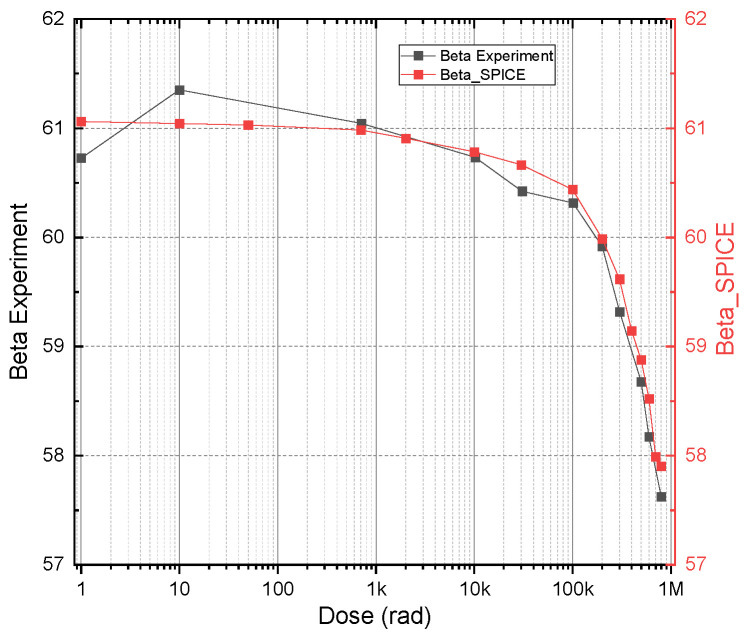
Dynamics of experimental and SPICE model maximum beta degradation.

The VBIC model [31] was chosen for its ease of integration into SPICE and its capability to approximate bipolar transistor behaviors accurately with minimal parameters. The extraction process is further streamlined due to the straightforward physical interpretation of its parameters, reducing both complexity and time investment. The estimated values of the parameters and their physical meaning are presented in Appendix A. The dynamics of maximum beta degradation and the convergence of experimental results and SPICE modeling are shown in Figure 8. The y-scale focuses on the β peak region and shows a very good fit between the experiments and simulations.

Based on the data in Appendix A, we note the dynamics of the main physical parameters, thanks to which it was possible to achieve a good match between the experimental results and the SPICE model.

The forward current gain β_F_ is found as follows:

$$BF\left(D\right)={BF}_{0}\cdot \exp\left(-1.15\times 10^{-5}\cdot D\right)$$

The saturation current *I_S_* is found as follows:

$$IS\left(D\right)={IS}_{0}\cdot exp\left(ln\left(10\right)\cdot 4.3\times 10^{-6}\cdot D\right)$$

The base resistance *R_B_* is found as follows:

$$RB(D)={RB}_{0}\cdot (1+2.86\times 10^{-6}\cdot D)$$

The forward transit time *TF* is found as follows:

$$TF(D)={TF}_{0}\cdot (1+2.14\times 10^{-6}\cdot D)$$

Parameters with 0 index (*BF*_0_, *IS*_0_, *RB*_0_, *TF*_0_) are initial values before the exposure.

*D*—Number for the total dose in krad_(Si)_.

### 3.3. Inverter Results

Due to experimental imperfections, data could only be obtained up to 600 krad_(Si)_ on the same wafer as the transistor presented above.

A more detailed look at the experimental results and the physical mechanisms of degradation under radiation is presented in [9]. The measurement method and control of the central point of the “butterfly” were described in [32,33].

**Figure 9:**
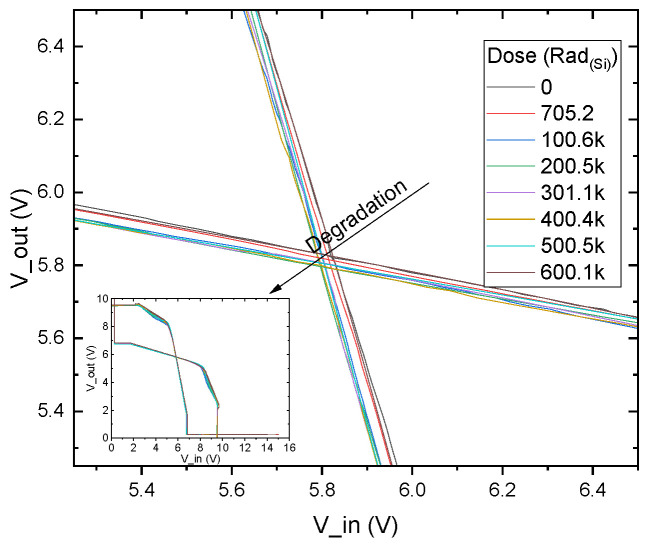
Inverter transfer characteristic degradation with radiation dose. The figure shows the central cross-cover of the transfer characteristics—input voltage versus output voltage. The inset shows the full characteristics.

**Figure 10:**
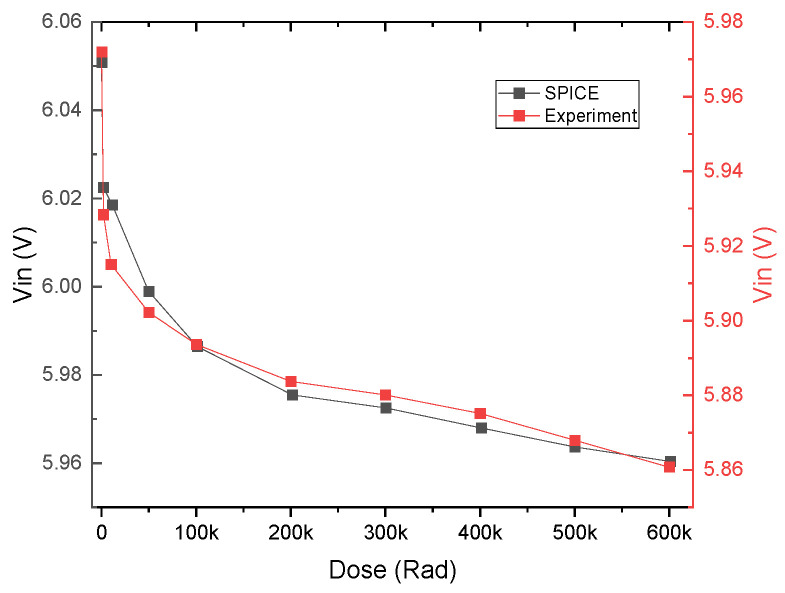
Inverter degradation with radiation dose: experimental vs. SPICE model.

Figure 9 and Figure 10 show the character of the inverter’s degradation and the convergence of experimental results with the SPICE model.

Approximation of the model results showed the following.

For the transistor:

• $BF(D)={BF}_{0}\cdot exp(-4.21\times 10^{-6}\cdot D)$
• $IS(D)=\;{IS}_{0}\times \;3.75{\times 10}^{-46}\cdot exp\;(1.47\times 10^{-5}\cdot D)$
• $RB(D)=\;{RB}_{0}+0.221\cdot D$
• $TF(D)={TF}_{0}\cdot (1+2.14\times 10^{-6}\cdot D)$

For the diode:

• $ISdiode\left(D)={3.75\times 10}^{-46}\cdot \;exp\right.(1.47\times 10^{-5}\cdot D)$
• N_diode_(D) = 1.55

Within the investigated range up to 600 krad_(Si)_, the inverter maintains acceptable operation: the switching midpoint only shifts slightly, and both high- and low-level plateaus remain distinct. Similar dose thresholds for silicon circuits are typically reported in the range of 10–100 krad_(Si)_ [34,35]. To define a quantitative limit for inverter usefulness, we introduce a failure criterion corresponding to a 30% reduction in the switching midpoint with respect to its initial value.

Extrapolation of the dose-dependent by presented SPICE model gives a value to failure of the device in the order of approximately 16 Mrad_(Si)_.

Analysis shows that the radiation degradation of the SiC-based inverter is primarily associated with changes in the BJT. A decrease in the current gain (BF) and an increase in the base resistance (RB) and transit time (TF) are the main factors that degrade the switching speed and reliability of the circuit. At the same time, the diode part of the inverter shows high radiation resistance, which makes it a more predictable element. This data is critically important for creating an accurate SPICE model capable of predicting the inverter’s behavior under radiation conditions and, thus, contributing to the development of radiation-hardened integrated circuits.

## 4. Discussion

Analysis of the extracted SPICE parameters revealed clear signs of degradation as a function of the accumulated radiation dose, indicating damage occurring both in the bulk semiconductor and at the device interface. The observed changes were categorized into three primary degradation mechanisms, each impacting different aspects of inverter performance. We will first discuss the different effects of gamma rays on individual BJTs and then conclude on the total degradation effects of the whole inverter circuit.

Deterioration of Amplification and Switching Properties

The most significant changes were observed in parameters governing current amplification and switching speed. The forward current gain (β_F_) was found to decrease with increasing dose, which is a direct physical consequence of an increased number of recombination centers within the base region. These radiation-induced defects shorten the minority carrier lifetime, consequently reducing the carrier transfer coefficient and leading to a substantial decrease in amplification efficiency. Simultaneously, the transistor’s switching speed was affected by a linear increase in both base resistance (RB) and forward transit time (TF) with increasing radiation dose. An increase in RB slows down the charging and discharging of junction capacitances, while an increase in TF is directly linked to the reduced carrier lifetime. The combined effect of these factors leads to a pronounced decrease in the overall switching speed of the transistor, limiting its applicability in high-frequency circuits.

Increase in Leakage Currents and Power Consumption

Degradation was also evident in parameters related to leakage currents. The saturation current (IS) showed moderate fluctuations with dose. In some cases, IS increased, likely due to the generation of radiation-induced traps in the p-n junctions, leading to higher leakage currents and degraded energy efficiency. Conversely, a decrease in IS was observed in other instances, which may be attributed to a complex redistribution of internal fields. The emission coefficient (NF), indicating the ideality of the bulk emitter junction, increased significantly with dose, suggesting a strong deviation from ideal carrier transport caused by enhanced recombination via the Shockley–Read–Hall (SRH) mechanism in the base region [29].

Impact of Surface-Related Degradation

Even more pronounced changes were observed in parameters related to surface effects: ISE and NE. The surface generation current (ISE), which was negligibly small in the unirradiated state (ISE ≈ 1 × 10^−15^ A), increased by more than three orders of magnitude at doses above 400 krad, reaching approximately 1 × 10^−11^ A. Simultaneously, the surface emission coefficient (NE) sharply increased, exceeding values of 90. These dramatic shifts indicate a highly non-ideal and severely degraded surface recombination process, directly pointing to significant degradation of the SiC/SiO_2_ interface [5,29,30] and underscoring the critical vulnerability of the device’s surface to radiation damage. Similar surface-related degradation phenomena have been reported for SiC/SiO_2_ interfaces under TID stress from charged particles as protons and ions, which are common in near-Earth space [36,37].

The physical interpretations of the steep increase in ISE and NE with radiation dose are consistent with established mechanisms of interface degradation in wide-bandgap semiconductors. Gamma irradiation induces positive fixed charges and interface traps both in the gate oxide and at the SiC/SiO_2_ interface. These trapped charges distort the local electric field near the base–emitter junction, which in turn enhances recombination through interface states.

The buildup of positive charge in the oxide attracts electrons and alters the electric field, increasing leakage and generation currents, especially under low-injection conditions. The deviation from exponential *I_c_* behavior at low *V_be_* values confirms the presence of shallow-level traps and a high density of interface states. These effects are accurately captured by the sharp increases in *I_SE_* and *N_E_*, making these parameters reliable markers of interface degradation.

Degradation of the full SiC-based inverter is a direct consequence of the accumulated changes in the characteristics of its transistor bases. While the diode in the circuit demonstrates high radiation hardness (its ideality coefficient and junction capacitance remain stable), the degradation of the BJT determines the overall performance reduction in the inverter. In multi-stage circuits where transistors operate in series or parallel configurations, the degradation of each individual component cumulatively affects the behavior of the entire circuit.

The increase in base resistance (RB) and forward transit time (TF) in each transistor leads to a cumulative increase in switching delays, which critically reduces the maximum operating frequency of the entire inverter. The significant decrease in forward current gain (BF) impairs the current-amplification properties, which can lead to incomplete saturation of transistors, distortion of output logic levels, and a reduction in the noise margin. Furthermore, the increase in leakage current (IS) in each transistor of the circuit leads to a cumulative increase in the total static power loss of the entire inverter, reducing its overall energy efficiency.

Overall, the changes observed in the SPICE model parameters form a direct and physically meaningful link between the radiation-induced effects at the material level and the observable behavior at the circuit level. This enables more accurate modeling and reliability assessment of SiC BJTs operating in radiation-intensive environments.

However, it is important to acknowledge the limitations of the proposed SPICE model. The parameters were extracted from DC characteristics and, while accurately reflecting the TID effects on circuit performance metrics like current gain, the model does not explicitly account for two important factors:

First, the model is a “DC-centric” characterization and does not directly incorporate the dynamic effects or other transient phenomena. While the increase in the mentioned parameters predicts slower switching, a full model would require dynamic testing and more complex parameterization.

Second, the model is calibrated for a specific device geometry and fabrication process at KTH. While the physical mechanisms captured (bulk and surface recombination) are general, the precise dose-dependent expressions for BF, IS, RB, and TF are empirical and may require re-calibration for SiC BJTs from different fabs or with different designs/processing flows.

Despite these limitations, the model provides a highly practical, accurate, and efficient engineering tool for predicting the radiation hardness and reliability of SiC integrated TTL circuits for harsh environments.

## 5. Conclusions

In conclusion, we have developed and validated a SPICE model that accurately predicts the impact of gamma radiation on both individual integrated SiC BJTs and complex TTL inverters. The model’s robustness and predictive power have been confirmed through a comprehensive comparison with experimental data. This work represents a significant step forward in understanding the degradation mechanisms of SiC-based integrated circuits in radiation-intensive environments. It is important to note that the model successfully demonstrated its applicability and accuracy for the devices described in the article, while also laying a methodological foundation for future research.

The analysis confirms that the primary cause of performance and reliability degradation in SiC inverters under radiation is the complex, cumulative degradation of their transistor components. We have demonstrated that the key parameters of the BJT model, such as forward current gain (BF), base resistance (RB), and saturation current (IS), show a clear and predictable dependence on the total ionizing dose. These patterns underscore the critical necessity of creating highly accurate SPICE models that can adequately account for radiation-induced parameter changes.

The experimental data and their precise alignment with our SPICE models highlight the importance of meticulous parameter tuning for ensuring the reliable operation of transistors in such harsh conditions. This achievement not only provides a foundational tool for the design of robust, radiation-hardened integrated circuits but also paves the way for further refinements in modeling methodologies and their broader application. Our findings also convincingly demonstrate the clear advantage of SiC TTL circuits over current Si technology, which is a pivotal step toward enabling a new era of reliable electronics for the exploration of space and other extreme environments.

With the well-calibrated model presented here, the projected “killing” dose for the SiC TTL inverter—defined as a 30% reduction in the switching midpoint—is approximately 16 Mrad_(Si)_, i.e., orders of magnitude beyond typical silicon logic, which commonly exhibits functional degradation/failure in the 10–100 krad_(Si)_ range [33,34].

Comparable findings regarding silicon logic degradation have been reported in [34,35], underscoring the relative robustness of SiC designs.

## Figures and Tables

**Table 1:** Total ionizing dose benchmarks.

Environment	Duration	Typical TID (rad_(Si)_)	Est. Dose in SiC (rad_(SiC)_)
LEO (ISS)	1 year	~3000	~1500
GEO	5 years	~50,000	~25,000
Jupiter orbiter	1 year	~200,000	~100,000
Lunar Base	10 years	~15,000	~7500

## Data Availability

The original contributions presented in this study are included in the article. Further inquiries can be directed to the corresponding author.

## Appendix A

**Table A1:** Gummel transistor parameters as functions of radiation dose in rad.

SiC BJT SPICE Parameters								
Parameter	Models	Units	0	100 k	300 k	500 k	700 k	800 k
bf	Ideal maximum forward beta	-	75	38.49	25.37	21.47	19.75	18.17
is	Transport saturation current	[A]	3.75 × 10^−46^	9.42 × 10^−44^	2.98 × 10^−42^	1.19 × 10^−41^	2.37 × 10^−41^	4.72 × 10^−41^
vaf	Forward early voltage	[V]	600					
var	Reverse early voltage	[V]	45					
rb	Zero-bias base resistance	[Ohm]	1.40 × 10^4^	3.64 × 10^4^	5.04 × 10^4^	5.60 × 10^4^	5.88 × 10^4^	6.16 × 10^4^
TF	Forward transit time		1.05 × 10^−10^	2.31 × 10^−10^	3.10 × 10^−10^	3.41 × 10^−10^	3.57 × 10^−10^	3.73 × 10^−10^
vaf	Forward early voltage	[V]	600					
var	Reverse early voltage	[V]	45					
**Inverter**								
**Parameter**	**Models**	**Units**	**0**	**100 k**	**300 k**	**500 k**		**600 k**
bf	Ideal maximum forward beta	-	75	38.49	25.37	21.47		19.75
is	Transport saturation current	[A]	3.75 × 10^−46^	9.42 × 10^−44^	2.98 × 10^−42^	1.19 × 10^−41^		2.37 × 10^−41^
vaf	Forward early voltage	[V]	600					
var	Reverse early voltage	[V]	45					
rb	Zero-bias base resistance	[Ohm]	1.40 × 10^4^	3.64 × 10^4^	5.04 × 10^4^	5.60 × 10^4^		5.80 × 10^4^
TF	Forward transit time	-	1.05 × 10^−10^	2.31 × 10^−10^	3.10 × 10^−10^	3.41 × 10^−10^		3.57 × 10^−10^
vaf	Forward early voltage	[V]	600					
var	Reverse early voltage	[V]	45					
Diode_IS	Diode transport saturation current	[A]	3.75 × 10^−46^	9.42 × 10^−44^	2.98 × 10^−42^	1.19 × 10^−41^		4.72 × 10^−41^
Diode_N	Diode emission coefficient	-	1.55					
Diode_CJO	Diode zero-bias junction capacitance	[F]	8.00 × 10^−40^					

## References

[1] Tudisco S., Altana C., Amaducci S., Ciampi C., Cosentino G., De Luca S., La Via F., Lanzalone G., Muoio A., Pasquali G. (2024). Silicon Carbide devices for radiation detection: A review of the main performances. Nucl. Instrum. Methods Phys. Res. Sect. A Accel. Spectrometers Detect. Assoc. Equip..

[2] Shakir M., Hou S., Hedayati R., Malm B.G., Östling M., Zetterling C.-M. (2019). Towards silicon carbide VLSI circuits for extreme environment applications. Electronics.

[3] Oldham T.R., McLean F.B. (2003). Total ionizing dose effects in MOS oxides and devices. IEEE Trans. Nucl. Sci..

[4] Pease R.L. (2003). Total ionizing dose effects in bipolar devices and circuits. IEEE Trans. Nucl. Sci..

[5] Suvanam S.S., Lanni L., Malm B.G., Zetterling C.M., Hallén A. (2014). Effects of 3-MeV protons on 4H-SiC bipolar devices and integrated OR-NOR gates. IEEE Trans. Nucl. Sci..

[6] Suvanam S.S., Lanni L., Malm B.G., Zetterling C.M., Hallén A. (2017). Total dose effects on 4H-SiC bipolar junction transistors. Mater. Sci. Forum.

[7] Chulapakorn T., Sychugov I., Suvanam S.S., Linnros J., Primetzhofer D., Hallén A. (2017). Influence of swift heavy ion irradiation on the photoluminescence of Si-nanoparticles and defects in SiO_2_. Nanotechnology.

[8] Metreveli A., Hallen A., Sarcina I.D., Cemmi A., Scifo J., Verna A., Zetterling C.-M. (2023). In Situ Gamma Irradiation Effects on 4H-SiC Bipolar Junction Transistors. IEEE Trans. Nucl. Sci..

[9] Metreveli A., Cuong V., Kuroki S., Tanaka K., Zetterling C. (2024). Impact of interface oxide type on the gamma radiation response of SiC TTL ICs. Facta Univ.-Ser. Electron. Energet..

[10] Lee J.-Y., Shakti S., Cooper J. (2008). Demonstration Characterization of Bipolar Monolithic Integrated Circuits in 4H-SiC. IEEE Trans. Electron Devices.

[11] Nava F., Bertuccio G., Cavallini A., Vittone E. (2008). Silicon Carbide and Its Use as a Radiation Detector Material. Meas. Sci. Technol..

[12] Zhang Q., Liu Y., Li H., Wang J., Wang Y., Cheng F., Han H., Zhang P. (2024). A Review of SiC Sensor Applications in High-Temperature and Radiation Extreme Environments. Sensors.

[13] Schwank J.R., Ferlet-Cavrois V., Shaneyfelt M.R., Paillet P., Dodd P.E. (2003). Radiation effects in SOI technologies. IEEE Trans. Nucl. Sci..

[14] Ren Y., Zhu M., Dai X., Li L., Liu M. (2025). Overview of the Properties and Formation Process of Interface Traps in MOS and Linear Bipolar Devices. Micromachines.

[15] Lanni L., Ghandi R., Malm B.G., Zetterling C.-M., Ostling M. (2012). Design and Characterization of High-Temperature ECL-Based Bipolar Integrated Circuits in 4H-SiC. IEEE Trans. Electron Devices.

[16] Zetterling C.-M., Hallén A., Hedayati R., Kargarrazi S., Lanni L., Malm B.G., Mardani S., Norström H., Rusu A., Suvanam S.S. (2017). Bipolar integrated circuits in SiC for extreme environment operation. Semicond. Sci. Technol..

[17] Baccaro S., Cemmi A., Di Sarcina I., Ferrara G. Gamma irradiation Calliope Facility at ENEA—Casaccia Research Centre (Rome, Italy). ENEA Technical Report RT/2019/4/ENEA. https://iris.enea.it/bitstream/20.500.12079/6838/1/RT-2019-04-ENEA.pdf.

[18] MIL-STD-883L. TEST METHOD STANDARD MICROCIRCUITS. U.S.A. 2019. https://s3vi.ndc.nasa.gov/ssri-kb/static/resources/std883.pdf.

[19] MIL-STD-750. TEST METHOD STANDARD, ENVIRONMENTAL TEST METHODS FOR SEMICONDUCTOR DEVICES, 2017. https://nepp.nasa.gov/DocUploads/87F5C780-EF36-4B8F-A2D5AC676D5456BF/MIL-STD-750.pdf.

[20] Drennan J.E., Hamman D.J. (1971). Radiation Effects Design Handbook.

[21] Bellan D., Brandolini A., Gandelli A. Effects of ADC nonlinearities in sine-wave amplitude measurement. Proceedings of the 1998 IEEE International Conference on Electronics, Circuits and Systems. Surfing the Waves of Science and Technology (Cat. No.98EX196).

[22] Barnaby H.J., Smith S.K., Schrimpf R.D., Fleetwood D.M., Pease R.L. (2002). Analytical model for proton radiation effects in bipolar devices. IEEE Trans. Nucl. Sci..

[23] Hazdra P., Smrkovský P., Popelka S. (2021). Radiation Defects and Carrier Lifetime in 4H-SiC Bipolar Devices. Phys. Status Solidi A.

[24] Wang J., Tan J., Wing O. Theory of cross-coupled RF oscillator for multi- and quadrature-phase signal generation. Proceedings of the 2003 5th International Conference on ASIC Proceedings.

[25] Metreveli A., Hallén A., di Sarcina I., Cemmi A., Verna A., Zetterling C.M. (2024). The Impact of Gamma Irradiation on 4H-SiC Bipolar Junction Inverters under Various Biasing Conditions. Solid State Phenom..

[26] Buono B., Ghandi R., Domeij M., Malm G., Zetterling C.-M., Östling M. (2010). Influence of Emitter Width and Emitter–Base Distance on the Current Gain in 4H-SiC Power BJTs. IEEE Trans. Electron Devices.

[27] York B. Modeling BJTs in Multisim. UCSB, [Online]. https://www.mikrocontroller.net/attachment/168555/Modeling_BJTs_in_Multisim.pdf.

[28] McAndrew C.C., Seitchik J., Bowers D., Dunn M., Foisy M., Getreu I., McSwain M., Moinian S., Parker J., Roulston D. (1996). VBIC95, The Vertical Bipolar Inter-Company Model. IEEE J. Solid-State Circuits.

[29] Schrimpf R., Fleetwood D., Pease R.L., Tsetseris L., Pantelides S.T. (2008). Impact of Radiation-Induced Defects on Bipolar Device Operation. Defects in Microelectronic Materials and Devices.

[30] Suvanam S.S., Usman M., Gulbinas K., Grivickas V., Hallén A. (2013). A Comparison of Free Carrier Absorption and Capacitance Voltage Methods for Interface Trap Measurements. Mater. Sci. Forum.

[31] Vladimirescu A. (1994). The SPICE Book.

[32] Massobrio G., Antognetti P. (1993). Semiconductor Device Modeling with SPICE.

[33] Katz R., Swift G., Shaw D. (1995). Total dose responses of ACTEL 1020B and 1280A field programmable gate arrays. Proceedings of the RADECS ′95 Conference.

[34] Tu R., Lum G., Pavan P., Ko P., Hu C. Simulating total-dose radiation effects on circuit behavior. Proceedings of the 1994 IEEE International Reliability Physics Symposium.

[35] Pien C.F., Amir H.F., Salleh S., Muhammad A. (2010). Effects of Total Ionizing Dose on Bipolar Junction Transistor. Am. J. Appl. Sci..

[36] Siddiqui A., Usman M. (2021). Radiation tolerance comparison of silicon and 4H–SiC Schottky diodes. Mater. Sci. Semicond. Process..

[37] Liu C., Li X., Geng H., Zhao Z., Yang D., He S. (2010). Radiation effects on bipolar junction transistors induced by 25 MeV carbon ions. Nucl. Instrum. Methods Phys. Res. Sect. A.

